# Modulation of Theta-Band Local Field Potential Oscillations Across Brain Networks With Central Thalamic Deep Brain Stimulation to Enhance Spatial Working Memory

**DOI:** 10.3389/fnins.2019.01269

**Published:** 2019-11-26

**Authors:** Ching-Wen Chang, Yu-Chun Lo, Sheng-Huang Lin, Shih-Hung Yang, Hui-Ching Lin, Ting-Chun Lin, Ssu-Ju Li, Christine Chin-jung Hsieh, Vina Ro, Yueh-Jung Chung, Yun-Chi Chang, Chi-Wei Lee, Chao-Hung Kuo, Shin-Yuan Chen, You-Yin Chen

**Affiliations:** ^1^Department of Biomedical Engineering, National Yang Ming University, Taipei, Taiwan; ^2^The Ph.D. Program for Neural Regenerative Medicine, College of Medical Science and Technology, Taipei Medical University, Taipei, Taiwan; ^3^Department of Neurology, Hualien Tzu Chi Hospital, Buddhist Tzu Chi Medical Foundation, Hualien City, Taiwan; ^4^Department of Neurology, School of Medicine, Tzu Chi University, Hualien City, Taiwan; ^5^Department of Mechanical Engineering, National Cheng Kung University, Tainan, Taiwan; ^6^Department and Institute of Physiology, National Yang Ming University, Taipei, Taiwan; ^7^Taiwan International Graduate Program in Interdisciplinary Neuroscience, National Yang Ming University, Academia Sinica, Taipei, Taiwan; ^8^Department of Neurosurgery, Taipei Veterans General Hospital, Neurological Institute, Taipei, Taiwan; ^9^Department of Neurological Surgery, University of Washington, Seattle, WA, United States; ^10^Department of Neurosurgery, Hualien Tzu Chi Hospital, Buddhist Tzu Chi Medical Foundation, Hualien City, Taiwan; ^11^Department of Surgery, School of Medicine, Tzu Chi University, Hualien City, Taiwan

**Keywords:** central thalamus, deep brain stimulation, spatial working memory, synaptic plasticity, hippocampal theta oscillation

## Abstract

Deep brain stimulation (DBS) is a well-established technique for the treatment of movement and psychiatric disorders through the modulation of neural oscillatory activity and synaptic plasticity. The central thalamus (CT) has been indicated as a potential target for stimulation to enhance memory. However, the mechanisms underlying local field potential (LFP) oscillations and memory enhancement by CT-DBS remain unknown. In this study, we used CT-DBS to investigate the mechanisms underlying the changes in oscillatory communication between the CT and hippocampus, both of which are involved in spatial working memory. Local field potentials (LFPs) were recorded from microelectrode array implanted in the CT, dentate gyrus, cornu ammonis (CA) region 1, and CA region 3. Functional connectivity (FC) strength was assessed by LFP–LFP coherence calculations for these brain regions. In addition, a T-maze behavioral task using a rat model was performed to assess the performance of spatial working memory. In DBS group, our results revealed that theta oscillations significantly increased in the CT and hippocampus compared with that in sham controls. As indicated by coherence, the FC between the CT and hippocampus significantly increased in the theta band after CT-DBS. Moreover, Western blotting showed that the protein expressions of the dopamine D1 and α4-nicotinic acetylcholine receptors were enhanced, whereas that of the dopamine D2 receptor decreased in the DBS group. In conclusion, the use of CT-DBS resulted in elevated theta oscillations, increased FC between the CT and hippocampus, and altered synaptic plasticity in the hippocampus, suggesting that CT-DBS is an effective approach for improving spatial working memory.

## Introduction

Deep brain stimulation (DBS) is a well-established neurosurgical technique applied during treatment for movement and psychiatric disorders. A medical device known as a neurostimulator is involved, with which local stimulation is performed on patients through electrodes implanted in specific brain regions to send electrical impulses to particular brain targets (Jacobs et al., 2016; Kuhn and Volkmann, 2017). In clinical applications, DBS has been used to treat a variety of neurological disorders by targeting nuclei in different brain regions. For instance, patients with Parkinson’s disease (PD) demonstrated improved motor symptoms after the application of DBS in the subthalamic nucleus, which is a key node in the functional control of motor activity in basal ganglia (Benabid, 2003; Duncan et al., 2018). Also, after DBS in the nucleus accumbens (NAc) in patients with autism spectrum disorders, the patients’ social communication skills were enhanced, and decreased metabolism in the prefrontal and frontal cortex were observed through fluorodeoxyglucose-positron emission tomography (Park et al., 2016). Furthermore, patients with major depressive disorder (MDD) treated with DBS in medial forebrain bundle had exhibited an improvement in their depression score due to the DBS-induced modulation of the mesolimblic reward system (Temel et al., 2015).

Recently, it has been reported that DBS has the ability to activate local and network-wide electrical effects and modulate oscillatory activities (Chiken and Nambu, 2014; Herrington et al., 2016). Additionally, several studies have revealed that DBS may modulate local field potentials (LFPs) by phase synchronization and rhythmic oscillations (de Hemptinne et al., 2015; Lin et al., 2015; Stefani et al., 2018). In humans, beta LFP oscillations appear to be related to motor function and gamma LFP oscillations related to sensory perception (Luo and Guan, 2018). In PD patients, DBS in the basal ganglia has been shown to inhibit beta LFP oscillations in the motor cortex, thereby improving cortical functions (de Hemptinne et al., 2015). In the Tourette syndrome (TS) patients, DBS in the centromedian nucleus (CM) of thalamus has been shown to increase the gamma LFP oscillations in the CM and also to ameliorate the TS symptoms (Maling et al., 2012). Furthermore, theta and alpha LFP oscillations are associated with memory (Colgin, 2013) and cognitive function (Klimesch, 2012), respectively. In MDD patients, DBS in the ventral internal capsule/ventral striatum increases theta oscillations in the prefrontal cortex, leading to enhanced performance of cognitive control tasks (Widge et al., 2019). In PD patients, DBS of caudal, and rostral pedunculopontine nucleus has been shown to inhibit alpha oscillations and improve gait (Thevathasan et al., 2012).

A number of studies have attempted to enhance memory and cognitive function by stimulating different DBS targets in humans and rodents. In humans, stimulation of the entorhinal cortex served to enhance spatial memory and increase theta oscillations in the hippocampus (Suthana et al., 2012). In rodents, theta oscillations in the hippocampus were restored by stimulating the fornix, and spatial working memory task performance was improved (Bick and Eskandar, 2016). Also, after application of DBS in the infralimbic cortex fin rodents, the cognitive function and memory were improved, and theta oscillations in the hippocampus were restored (Cervera Ferri et al., 2016). An improvement in working memory was found after electrical stimulation of the central thalamus (CT) in object recognition memory tasks in rodents (Mair and Hembrook, 2008). Moreover, forniceal DBS in Rett syndrome mice rescued spatial learning and memory, and restored *in vivo* hippocampal synaptic plasticity and hippocampal neurogenesis (Hao et al., 2015).

Enhancements of cognitive function and memory have been indicated through synaptic plasticity modification induced by DBS in the CT (Lin et al., 2015; Tsai et al., 2016; Wang et al., 2017). Restoration of consciousness and enhancement of cognitive function were demonstrated in patients with traumatic brain injury and disorders of consciousness after the implication of CT-DBS (Shah and Schiff, 2010; Tabansky et al., 2014; Schiff, 2016; Carlton and Murad, 2018). The activation of *c-Fos* and Zif268 in the cortical region and hippocampus has been shown to be modulated by DBS in the CT, which significantly improved behavioral performance associated with cognitive memory function in rodents (Shirvalkar et al., 2006). Application of DBS promotes the release of striatal dopamine and hippocampal acetylcholine (Figee et al., 2014; Posporelis et al., 2018). In human studies, DBS targeting of the NAc was indicated to be related to the dopaminergic system in the striatum. Patients with obsessive-compulsive disorder treated with DBS in the NAc were found to exhibit an increase in the dopamine release in the striatum, further increasing dopamine neurotransmission, leading to an improvement in their clinical symptoms (Figee et al., 2014). DBS in the medial septum applied in an Alzheimer’s rodent models indicated an increase in acetylcholine release in the hippocampus and possible reversal of spatial memory impairments (Posporelis et al., 2018). Both dopaminergic and acetylcholine-mediated signaling are important for synaptic plasticity modification (Di Filippo et al., 2008).

The central thalamus comprises the central lateral nucleus, mediodorsal nucleus, parafascicular nucleus, CM and nucleus reuniens (Saalmann, 2014). In a previous study, the CT was revealed to play a critical role in the extra-hippocampal network in terms of spatial working memory consolidation (Lopez et al., 2009). The known anatomical projection of the CT includes the dentate gyrus (DG) (Shirvalkar et al., 2006), which is a subregion of the hippocampus. The hippocampus has been reported to receive dense innervation by cholinergic neurons, which serve to mediate the formation of memory; on the other hand, the acetylcholine has not only been shown to play a critical role in the hippocampus as a modulator of cognitive function, but has also aroused significant attention for its extensive effects on recovery of impaired memory (Haam and Yakel, 2017; Maurer and Williams, 2017). In rodent studies, an increase in acetylcholine in the DG was observed after the application of electrical stimulation to the hippocampus in intact mice, leading to an improvement in learning and memory performance (Matsuyama et al., 2000). A previous study showed that dopaminergic neurons innervate the hippocampus, and hippocampal dopamine signaling has been indicated as strongly involved in spatial memory and cognitive function (Edelmann and Lessmann, 2018). Rodent models that had received the neurosurgical treatment of electrical stimulation have been observed to demonstrate the activation of dopamine in hippocampus and enhancement of behavioral performance (Li et al., 2003). The hippocampus comprises two characteristic interlocking C-shaped layers of cells, including the cornu ammonis (CA) region 1 (CA1), CA region 3 (CA3), and DG, also known as the trisynaptic circuit (Yeckel and Berger, 1990). It has been suggested that the above-mentioned regions serve different roles and exhibit distinct functions in the mediation of memory; e.g., the projection of the DG to CA3, which is essential for the process of spatial information encoding (Kesner et al., 2004). In contrast, the function of CA3 involves the rapid acquisition of novel information (Kesner, 2007), whereas that of CA1 is associated with temporal pattern separation (Gilbert et al., 2001). Previous studies have indicated that lesions in the DG and CT may result in neural activity inhibition and spatial working memory impairment based on the observation of delayed match-to-position tasks (Mizumori et al., 1994; Kesner et al., 2004; Mair et al., 2011).

Although structural and functional connections between the CT and hippocampus have been reported, memory enhancement and oscillatory communication between the two regions remain largely unknown. In this study, we investigated changes in both LFP oscillations and functional connectivity (FC) among four specific brain regions, including the CT, CA1, CA3, and DG after the application of CT-DBS. In addition, a T-maze behavioral task was employed to evaluate the effect of CT-DBS on spatial working memory. We hypothesized that increased FC between the CT and hippocampus induced by CT-DBS indicates an alteration of structural neuroplasticity. Therefore, Western blotting was used to examine CT-DBS-induced protein expression changes in dopamine and acetylcholine receptors.

## Materials and Methods

### Animal Subjects and Grouping

In total, 30 male adult SD rats weighing between 250 and 350 g were used in this study. All rats were kept in an animal research facility under well-controlled laboratory conditions (12:12 light: dark cycle with lights kept on at 7 AM; 20°C ± 3°C) and fed *ad libitum*. All procedures followed the National Institute of Health’s guidelines for animal care and procedures and were approved by the Institutional Animal Care and Use Committee of Tzu Chi General Hospital (IACUC Approval No.: 106-35).

The rats were equally divided into three groups (*N* = 10 per group): sham controls (sham stimulation), DBS group and DBS *wo.* T-maze group. Rats in sham controls (sham stimulation) and DBS group with implanting a microelectrode array and then received the T-maze behavioral test following CT-DBS (or sham stimulation), which was used to compare the improvement in spatial working memory using CT-DBS. To exclude the effects of T-maze behavioral training on LFP oscillation changes, rats in the DBS *wo.* T-maze group only received the CT-DBS treatment without T-maze test, which confirm the presence of CT-DBS-evoked LFP oscillation changes in Supplementary Figure S1 and Supplementary Table S1 (see Supplementary Note S1).

**FIGURE 1:**
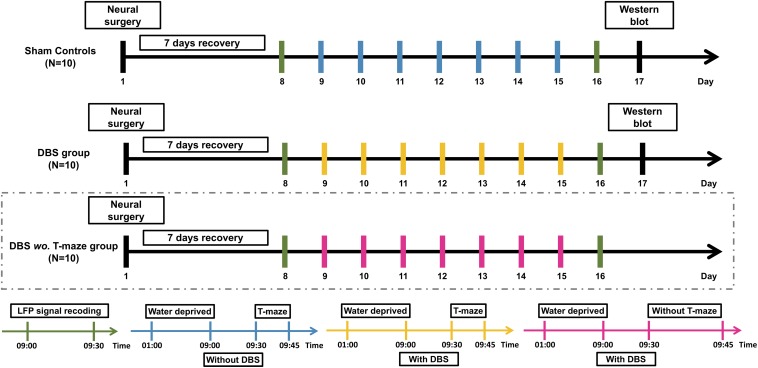
Experimental procedure of CT-DBS evaluation. Thirty rats were divided into sham controls (*N* = 10), DBS group (*N* = 10), and DBS *wo.* T-maze group (*N* = 10). After a 7-day surgical recovery, implanted rats in sham controls and DBS group received CT-DBS (or sham stimulation) and underwent T-maze behavioral task training and LFP recording. For confirmation of CT-DBS-induced dynamic changes in LFP oscillatory activity in different hippocampal regions, to exclude confounding effects of T-maze behavioral training, we added a group of DBS *without* T-maze task (DBS *wo.* T-maze group, marked by dashed box). LFP recording (green bar): LFP recording was performed twice, i.e., on the 8th day for baseline and before Western blotting on the 16th day. Sham CT-DBS (blue bar): rats were placed in a plastic cage without 30-min CT-DBS and then trained for the T-maze behavioral task for 7 days. For CT-DBS (yellow bar): rats were placed in the same plastic cage with 30-min CT-DBS and then trained for the T-maze behavioral task for 7 days. Rats in sham controls and DBS group were sacrificed for protein level analysis by Western blotting on the 17^th^ day (black bar).

The experimental timeline is shown in Figure 1. The rats were allowed to recover for 7 days following implantation before the start of CT-DBS (or sham stimulation). Implanted rats in sham controls and DBS group performed a 30-min LFP recording twice, i.e., on the 8^th^ and 16^th^ day at 9:00–9:30 AM. The first LFP recording was used to establish a baseline before starting the T-maze behavioral task, whereas the second recording was used to evaluate the altered LFP oscillations and LFP–LFP coherence between the two brain regions with CT-DBS. From the 9^th^ to 15^th^ day, each rat in sham controls and DBS group was placed in a plastic cage for 30 min/day (9:00–9:30 AM) with/without CT-DBS, and then the rats performed the T-maze behavioral task (9:30–9:45 AM). Following the second LFP recording, rats in sham controls and DBS group were sacrificed for protein analysis by Western blotting on the 17^th^ day. For only demonstration of the CT-DBS-evoked LFP oscillation changes in the DBS *wo.* T-maze group, all aspects of the experimental procedures were the same as described for sham controls and DBS group, except that the rats were excluded from T-maze behavioral testing and the Western blotting.

### Animal Surgical Procedures for Neural Implantation

Surgeries were performed on both groups. The rats were anesthetized with intramuscular administration of 40 mg/kg zolazepam and tiletamine (Zoletil 50, Virbac, Corros, France) and 8 μg/kg dexmedetomidine hydrochloride (Dexdomitor^®^, Pfizer Inc., New York, NY, United States).

**FIGURE 2:**
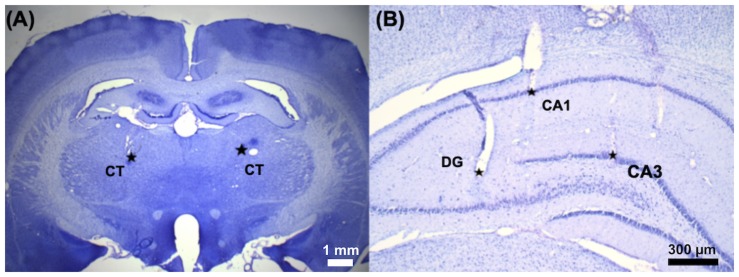
The implantation sites of the microelectrode array was confirmed by Nissl staining. A 16-channel stainless microwire electrode array was used to perform CT-DBS and multi-site LFP recordings. **(A)** The representative coronal brain slice shows two small electrolytic lesions made around the tip of the electrode by passing 50 μA DC for 30 s in the bilateral CT (AP, −2.7 mm; ML, ±1.6 mm; and DV, 5.5 mm) marked with two star-symbols (^∗^). **(B)** Three electrolytic lesions marked with three star-symbols (^∗^): left hippocampal DG (AP, −4.0 mm; ML, −2.0 mm; and DV, 3.6 mm), CA1 (AP, −4.0 mm; ML, −2.4 mm; and DV, 1.9 mm), and CA3 (AP, −4.0 mm; ML, −3.0 mm; and DV, 1.9 mm).

A 16-channel stainless microwire electrode array (product #M415050, 20-μm diameter, California Fine Wire Co., Grover Beach, CA, United States) was implanted into the bilateral CT [anterior/posterior (AP): −2.7 mm, medial/lateral (ML): ±1.6 mm, dorsal/ventral (DV): 5.5 mm], DG (AP: −4.0 mm, ML: ±2.0 mm, DV: 3.6 mm), CA1 (AP: −4.0 mm, ML: ±2.4 mm, DV: 1.9 mm), and CA3 (AP: −4.0 mm, ML: ±3.0 mm, DV: 1.9 mm), and each brain region contained two channels as shown in Figure 2. A stainless screw (BiFu Screw Parts Co., Ltd., Hsinchu, Taiwan) was secured to the skull over the cerebellum using dental cement (Coltene/Whaledent Inc., Cuyahoga Falls, OH, United States) as a reference electrode. After 7-day surgical recovery, the implanted rats received bilateral CT-DBS, LFP recordings were performed in the bilateral brain regions (CT, DG, CA3, and CA1), and the T-maze behavioral task was performed.

### LFP Recordings and Data Analysis

Local field potentials were bilaterally recorded in the CT, DG, CA1, and CA3 to investigate changes in neural oscillations and FC using the Cerebus data acquisition system (Blackrock Microsystems LLC, Salt Lake, UT, United States). The sampling rate was 1 kHz, and the signal was bandpass analog filtered at cut-off frequencies of 0.3 and 250 Hz. Data analysis was post-processed with MATLAB (R2018b, MathWorks Inc., Natick, MA, United States). The comparison of LFP oscillations and coherence between the two groups were further performed.

Power spectral density (PSD) was calculated using the LFP data for delta (1–4 Hz), theta (4–7 Hz), alpha (7–13 Hz) and beta (13–20 Hz) bands using Welch’s method (see Supplementary Note S2). Then, the PSD results for each frequency band were normalized using the following formula:

(1) $$P\,S\,D\%=\frac{P\,S\,D^{p\,o\,s\,t-t\,r\,e\,a\,m\,e\,n\,t}}{P\,S\,D^{b\,a\,s\,e\,l\,i\,n\,e}}\times 100\%$$

where *PSD*^*baseline*^and *PSD*^*post*−*treatment*^ were obtained from LFP PSD for each frequency band before (baseline) and after CT-DBS treatment or sham stimulation, respectively, in the CT, DG, CA1, and CA3.

To quantify the FC changes induced by CT-DBS, coherence (magnitude-squared coherence) was used to measure of linear association between two LFPs between two brain regions as a function of frequency as shown in the following equation:

(2) $$C\,o\,h_{r\,e\,g\,i\,o\,n\,A,B}\,\left(f\right)=\frac{{\left|P\,S\,D_{r\,e\,g\,i\,o\,n\,A,B}\,\left(f\right)\right|}^{2}}{P\,S\,D_{r\,e\,g\,i\,o\,n\,A,A}\,\left(f\right)\times P\,S\,D_{r\,e\,g\,i\,o\,n\,B,B}\,\left(f\right)}$$

where *PSD*_*regionA*,*A*_(*f*) and *PSD*_*regionB*,*B*_(*f*)represent the averages of the spectral powers of the LFP time series of region A and region B, respectively. *PSD*_*regionA*,*B*_(*f*) is the average cross-spectral power. The magnitude-squared coherence values for the two brain regions were computed using Welch’s method, a modified periodogram method. The magnitude-squared coherence estimate is a function of frequency with values ranging from 0 to 1, where a coherence of 0 indicates that the LFPs are unrelated and coherence of 1 indicates that the LFPs have a constant phase relationship. Data were analyzed offline using the custom-built MATLAB software (R2018b, MathWorks Inc., Natick, MA, United States). Magnitude-squared coherence measurement parameters include coherence frequency segment size (5,000 data points), Hanning window overlap (50%) and tapering, and sampling rate (1 kHz).

To compare the FC changes caused by CT-DBS, the measured FC between the distinct brain regions was normalized to the percentage coherence (*Coh*%), which was calculated using the post-treatment coherence by dividing the baseline coherence as shown in the following equation:

(3) $$C\,o\,h_{\left(r\,e\,g\,i\,o\,n\,A-r\,e\,g\,i\,o\,n\,B\right)}\%=\frac{C\,o\,h_{\left(r\,e\,g\,i\,o\,n\,A-r\,e\,g\,i\,o\,n\,B\right)}^{p\,o\,s\,t-t\,r\,e\,a\,t\,m\,e\,n\,t}}{C\,o\,h_{\left(r\,e\,g\,i\,o\,n\,A-r\,e\,g\,i\,o\,n\,B\right)}^{b\,a\,s\,e\,l\,i\,n\,e}}\times 100\%$$

where $C\,o\,h_{\left(r\,e\,g\,i\,o\,n\,A-r\,e\,g\,i\,o\,n\,B\right)}^{b\,a\,s\,e\,l\,i\,n\,e}$ and $C\,o\,h_{\left(r\,e\,g\,i\,o\,n\,A-r\,e\,g\,i\,o\,n\,B\right)}^{p\,o\,s\,t-t\,r\,e\,a\,t\,m\,e\,n\,t}$ were the chosen coherences between the two brain regions before (baseline) and after CT-DBS (or sham stimulation), respectively.

### Bilateral CT-DBS Protocol

The rats in the DBS group received 30-min bilateral CT-DBS in a plastic cage of 30-cm diameter and 38-cm height and were then trained for the T-maze behavioral task once a day for 7 days. Bipolar electrical stimulation with a pulse width of 25 μs/phase was administered to the bilateral CT using an isolated stimulator (Model 2100, A-M System Inc., Sequim, WA, United States). The intensity of the electrical stimulation was 250 μA at a frequency of 100 Hz. The sham controls were placed in the same plastic cage without CT-DBS for 30 min and then trained for the T-maze behavioral task once a day for 7 days.

### T-Maze Behavioral Task

The T-maze was mainly prepared using polyvinyl chloride plastic. The maze comprised an approach alley (90 cm × 10 cm, height: 10 cm) and two goal arms (50 cm × 10 cm, height: 10 cm) covered with a T-shaped transparent polymethyl methacrylate sheet to prevent the rats from slipping off the maze. Animal movement was recorded with a video camera (NeuroMotive^TM^, Blackrock Microsystems LLC, Salt Lake, UT, United States) positioned above the T-maze, and behavioral performance was analyzed by an open-source toolbox (Ben Shaul, 2017).

In this study, each rat underwent 5 trials daily in the T-maze behavioral tasks from the 9^th^ to 15^th^ day after 30-min CT-DBS or sham stimulation. Each trial was measured in 60 s, followed by a 30–90 s inter-trial interval for resting. The T-maze was wiped with alcohol between trials to remove any olfactory cues. For each trial, each rat was placed at the base of the T-maze and rewarded with water at one end of the goal arm. In this study, the water reward was always placed in the same goal arm. To evaluate the effects of CT-DBS on the behavioral performance in the T-maze behavioral task, the latency time to reach the water reward placed at the end of the goal arm and spatial working memory index (SWMI) (Bezu et al., 2017) was calculated:

(4) $$SWMI\left(\%\right)=\frac{n\,u\,m\,b\,e\,r\,o\,f\,c\,o\,r\,r\,e\,c\,t\,c\,h\,o\,i\,c\,e\,s}{n\,u\,m\,b\,e\,r\,o\,f\,t\,o\,t\,a\,l\,t\,r\,i\,a\,l\,s}\times 100\%$$

### Western Blotting

The DG, CA1, and CA3 were dissected from the brain tissues of the 10 study rats. Protein samples were extracted in ice-cold lysis buffer (50 mM Tris–HCl, pH = 7.5, 0.3 M sucrose, 0.5 mM EDTA, 2 mM sodium pyrophosphate, 1 mM sodium orthovanadate, 1 mM PMSF, 20 μg/mL leupeptin, and 4 μg/mL aprotinin) and then separated (30 μg) by SDS-PAGE. Gels were then transferred onto polyvinylidene difluoride membranes (Millipore, Billerica, MA, United States). The membranes were hybridized with the anti-dopamine D2 receptor (DRD2, 1:1000 dilution; ADR-002-50UL, Alomone Labs, Jerusalem, Israel) or anti-dopamine D1 receptor (DRD1, 1:1000 dilution; DR001AN03, Alomone Labs, Jerusalem, Israel) antibodies or anicotinic acetylcholine receptor alpha 4 (α4-nAChR, 1:1000 dilution; ANC-004-50UL, Alomone Labs, Jerusalem, Israel) antibodies. Next, the membranes were washed and incubated with HRP-conjugated goat anti-rabbit IgG antibody (1:1000 dilution; Jackson ImmunoResearch Inc., West Grove, PA, United States) and developed using the Luminata Forte Western HRP substrate (Millipore, Billerica, MA, United States). Images were recorded using a luminescence imaging system (LAS-4000, Fujifilm, Tokyo, Japan), and a gel analysis plug-in for the *ImageJ* software^1^ (ver. 1.47, National Institutes of Health, Bethesda, MD, United States) was used to quantify the intensity of the protein bands.

### Statistical Analysis

The normalized percentage of LFP PSD and FC analyses with the coherence between brain region pairs were compared before and after CT-DBS (or sham stimulation) by non-parametric statistical analysis, Wilcoxon signed-rank test, in each frequency band and FC for the two groups. In addition, the behavioral performances in terms of latency time and SWMI as well as protein expression levels of DRD1, DRD2, and α4-nAChR were analyzed by Wilcoxon two-sample *t*-test for comparing the differences between the two groups. A probability value of <0.05 was used as the criterion for determining statistical significance. All data are presented as the mean ± standard error of the mean (SEM). All statistical analyses mentioned above were performed using SPSS version 20.0 (SPSS Inc., Chicago, IL, United States) and their corresponding powers and effect sizes (Rosnow and Rosenthal, 1996; Rosnow et al., 2000) were determined using open source toolbox, G^∗^Power (version 3, Institut fürExperimentelle Psychologie, Dusseldorf, Germany) (Cacioppo et al., 2007; Faul et al., 2007), and Cohen’s *d* equation (Shavelson, 1988; Cohen, 1992), respectively. According to the results of power analyses and effect size test, sufficient statistical powers (>0.8) and medium to large effect sizes were shown in Supplementary Tables S2–S5 (see Supplementary Note S3).

## Results

### Behavioral Task Performance: Sham Controls vs. DBS Group

**FIGURE 3:**
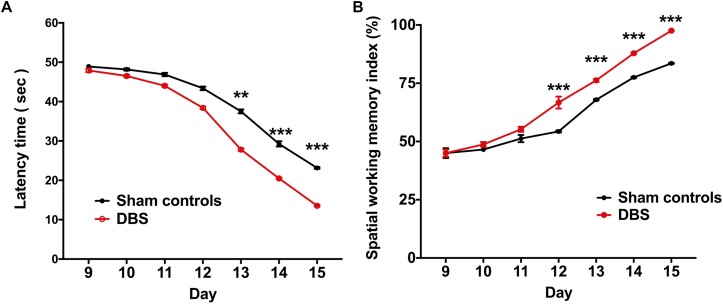
Comparsion of behavioral performances in the T-maze behavioral task between the groups following CT-DBS (or sham stimulation). **(A)** Curves of latency time (s) to reach the correct T-maze goal arm was plotted against the sessions, i.e., one session/day and five trials/session. There were significantly shorter learning periods found in the DBS group from the 13^th^ to 15^th^ day compared with those in the sham controls during T-maze behavioral task training. **(B)** SWMI showed the percentage of the mean correct ratio to reach the correct T-maze goal arm against the sessions as **(A)**. SWMI also was found to significantly increase from the 12^th^ to 15^th^ day in the DBS group. ^∗∗^, and ^∗∗∗^ indicate significant differences in terms of latency time and SWMI with *p* < 0.01 and *p* < 0.001, respectively, analyzed by Wilcoxon two-sample *t*-tests (mean ± SEM).

In the T-maze behavioral task, the rats were required to explore the routes for the water reward, and the behavioral performance indicators, i.e., latency time and SWMI, were analyzed to compare the CT-DBS effects on behavioral performance between the two groups. Latency time was significantly shorter to reach the criterion from the 13^th^ to 15^th^ day in the DBS group [13^th^ day: 27.81 ± 0.29 s (^∗∗^*p* = 0.00658), 14^th^ day: 20.48 ± 0.23 s (^∗∗∗^*p* = 0.00053), and 15^th^ day: 13.54 ± 0.25 s (^∗∗∗^*p* = 0.00075)] than that in the sham controls (13^th^ day: 37.51 ± 0.47 s, 14^th^ day: 29.26 ± 0.69 s, 15^th^ day: 23.14 ± 0.21 s) as shown in Figure 3A. As shown in Figure 3B, the SWMI values were significantly higher from the 12^th^ to 15^th^ day in the DBS group [12^th^ day: 66.72 ± 2.57% (^∗∗∗^*p* = 0.00045), 13^th^ day: 76.24 ± 0.65% (^∗∗∗^*p* = 0.00057), 14^th^ day: 87.87 ± 0.46% (^∗∗∗^*p* = 0.00036), and 15^th^ day: 97.56 ± 0.14% (^∗∗∗^*p* = 0.00029)] than those in the sham controls (12^th^ day: 54.33 ± 0.43%, 13^th^ day: 67.91 ± 0.32%, 14^th^ day: 77.50 ± 0.34%, and 15^th^ day: 83.54 ± 0.21%).

### Neural Oscillation: Before (Baseline) vs. After CT-DBS (or Sham Stimulation)

**FIGURE 4:**
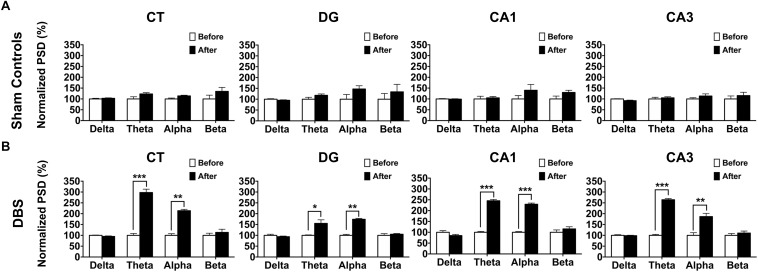
Comparsion of LFP oscillatory changes before (baseline) and after CT-DBS (or sham stimulation) in both groups. The normalized percentage of the LFP PSD was calculated as the ratio of the original PSD before CT-DBS or sham stimulation (baseline) to that after CT-DBS or sham stimulation in the CT, DG, CA1, and CA3. **(A)** In the sham controls, there were no significant differences in terms of delta, theta, alpha and beta bands at each site. **(B)** In the DBS group, LFP PSD showed significant enhancements for the theta and alpha bands in the CT, DG, CA1, and CA3 following CT-DBS. ^∗^, ^∗∗^, and ^∗∗∗^ indicate significant differences in terms of PSD with *p* < 0.05, *p* < 0.01, and *p* < 0.001, respectively, analyzed by Wilcoxon signed-rank tests (mean ± SEM).

Neural oscillations in the studied brain regions after CT-DBS may be directly associated with the enhancement in T-maze behavioral task performance. To evaluate this further, LFPs were also recorded in the CT, CA1, CA3, and DG. The detailed PSD traces of the sham controls and DBS group are presented in Supplementary Figure S2 (Supplementary Note S2). Furthermore, LFP oscillations were examined in the alpha, beta, delta, and theta bands. In Figure 4A, no significant differences were found in terms of the frequency bands of LFP oscillations in the four brain regions in the sham controls (sham stimulation) between before (baseline) and after the T-maze behavioral task (Wilcoxon signed-rank test). In the DBS group, there were significant increases in the LFP theta- and alpha-band oscillations in the CT [theta: 297.68 ± 15.58% (^∗∗∗^*p* = 0.00034) and alpha: 213.79 ± 6.30% (^∗∗∗^*p* = 0.00025)], DG [theta: 155.01 ± 15.85% (^∗^*p* = 0.03572) and alpha: 174.20 ± 4.17% (^∗∗∗^*p* = 0.00037)], CA1 [theta: 245.56 ± 5.89% (^∗∗∗^*p* = 0.00041) and alpha: 229.59 ± 5.65% (^∗∗∗^*p* = 0.00036)], and CA3 [theta: 264.50 ± 5.57% (^∗∗∗^*p* = 0.00024) and alpha: 186.75 ± 13.59%, (^∗∗^*p* = 0.00307)] following the T-maze behavioral task compared with the baseline values as shown in Figure 4B.

### Brain Connectivity: Before (Baseline) vs. After CT-DBS (or Sham Stimulation)

**FIGURE 5:**
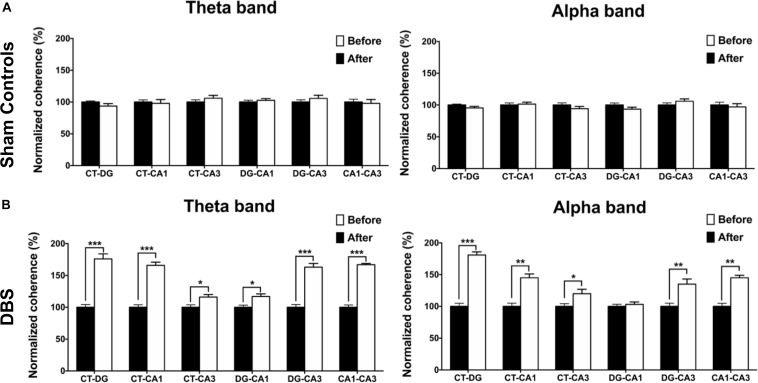
Statistical comparison of FC changes before (baseline) and after CT-DBS (or sham stimulation). FC analyses were used to estimate the normalized coherence between the pairs of brain regions at LFP recording sites (CT, DG, CA1, and CA3) in the two group. **(A)** The strengths of theta- and alpha-band coherences in FC showed no significant differences before and after sham stimulation in the sham controls. **(B)** In DBS group, FC strengths between the brain region pairs CT–DG, CT–CA1, CT–CA3, DG–CA1, DG–CA3, and CA1–CA3 significantly increased with theta-band coherences following 7-day CT-DBS. Further, the FC strengths between the brain region pairs CT–DG, CT–CA1, CT–CA3, DG–CA3, and CA1–CA3 showed significant increases for alpha-band coherences following 7-day CT-DBS. ^∗^, ^∗∗^, and ^∗∗∗^ indicate significant differences in terms of coherence with *p* < 0.05, *p* < 0.01, and *p* < 0.001, respectively, relative to CT-DBS (baseline), analyzed by Wilcoxon signed-rank tests (mean ± SEM).

To compare CT-DBS effects on brain connectivity between groups, LFP–LFP coherences between brain region pairs were used to perform FC analyses in the two groups. As shown in Figure 5A, the sham controls were not significantly different from the DBS group in terms of the theta- and alpha-band FC (Wilcoxon signed-rank test). As seen in Figure 5B, the DBS group showed significant increases in the theta-band FC strength for *Coh*_*(CT–DG)*_[176.02 ± 8.03% (^∗∗∗^*p* = 0.00048)], *Coh*_*(CT–CA1)*_ [166.14 ± 5.19% (^∗∗∗^*p* = 0.00078)], *Coh*_*(CT–CA3)*_ [116.42 ± 4.10% (^∗^*p* = 0.02407)], *Coh*_*(DG–CA1)*_ [117.28 ± 4.27% (^∗∗^*p* = 0.00792)], *Coh*_*(DG–CA3)*_ [163.76 ± 6.37% (^∗∗∗^*p* = 0.00038)], and *Coh*_*(CA1–CA3)*_ [167.63 ± 2.12% (^∗∗∗^*p* = 0.00054)] following the T-maze behavioral task. In addition, the alpha-band FC strength for *Coh*_*(CT–DG)*_ [181.49 ± 5.12% (^∗∗∗^*p* = 0.00057)], *Coh*_*(CT–CA1)*_ [145.13 ± 6.10% (^∗∗∗^*p* = 0.00042)], *Coh*_*(CT–CA3)*_ [120.46 ± 7.05% (^∗^*p* = 0.03104)], *Coh*_(*DG*−*CA*3)_ [135.81 ± 8.54% (^∗∗^*p* = 0.002537)] and *Coh*_*(CA1–CA3)*_ [145.23 ± 4.76% (^∗∗∗^*p* = 0.00067)] significantly increased in the DBS group. However, there were non-significant increases in the beta- and delta-band FC strengths in both groups as shown in Supplementary Figure S3 (see Supplementary Note S4).

### Western Blotting: Sham Controls vs. DBS Group

**FIGURE 6:**
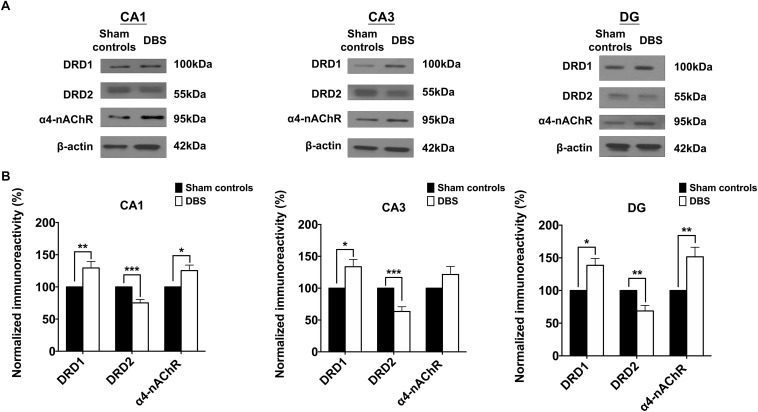
Comparsion of the two groups based on Western blotting results of the protein expressions of the DRD1, DRD2, and α4-nAChR receptors in the CA1, CA3, and DG. **(A)** The protein expressions of the DRD1, DRD2, and α4-nAChR receptors in the CA1, CA3, and DG in both sham controls and DBS group. **(B)** The mean normalized protein expression levels of the DRD1, DRD2, and α4-nAChR receptors in the CA1, CA3, and DG in the two groups. The CA1, CA3, and DG in the DBS group show significant increases in the protein expression levels of the DRD1 receptor but significant decreases in the levels of the DRD2 receptor compared with those in the sham controls. The CA1 and DG in the DBS group exhibited significant increases in the protein expression levels of the α4-nAChR receptor compared with those in the sham controls. ^∗^, ^∗∗^, and ^∗∗∗^ indicated significant differences in terms of protein expression with *p* < 0.05, *p* < 0.01 and *p* < 0.001, respectively, relative to the sham controls, analyzed by Wilcoxon two-sample *t*-tests (mean ± SEM).

To confirm the CT-DBS-induced synaptic plasticity changes in the hippocampus, we evaluated the expressions of DRD1, DRD2, and α4-nAChR receptors in CA1, CA3 and DG, which are known to play key roles in synaptic plasticity. The results were calculated as the ratio of DRD1, DRD2 and α4-nAChR to GAPDH. GAPDH levels were consistent across both groups. Our results demonstrated that significantly higher protein expressions of DRD1 and α4-nAChR and lower protein expression of DRD2 were associated with CT-DBS in the DBS group compared with those in sham controls in CA1, CA3, and DG (Figure 6A).

Statistical analyses further revealed that the normalized protein expression levels of DRD1 in CA1, CA3 and DG were significantly higher [CA1: 129.44 ± 10.03% (^∗∗^*p* = 0.00683), CA3: 133.67 ± 11.45% (^∗^*p* = 0.04702), DG: 138.55 ± 10.43% (^∗^*p* = 0.02048)], α4-nAChR [CA1: 125.34 ± 8.59% (^∗^*p* = 0.02607), DG: 151.64 ± 114.52% (^∗∗^*p* = 0.00760)] in the DBS group than in the sham controls (Figure 6B). Moreover, in the DBS group, CA1, CA3, and DG showed significantly lower protein expression levels of DRD2 [CA1: 75.15 ± 5.26% (^∗∗∗^*p* = 0.00034), CA3: 63.42 ± 7.44% (^∗∗∗^*p* = 0.00039), DG: 68.66 ± 8.42% (^∗∗^*p* = 0.00435)] than in the sham controls. The results demonstrated the regulation of DRD1, DRD2, and α4-nAChR expression in the CA1, CA3, and DG with CT-DBS treatment.

## Discussion

### CT-DBS Increased Theta Oscillations Associated With Spatial Working Memory as the Biomarker

In this study, CT-DBS enhanced both theta and alpha oscillations in the CT, CA1, CA3, and DG and improved T-maze behavioral task performance in a rat model related to spatial working memory. In a human study, hippocampal theta oscillation was related to spatial working memory or episodic memory (Klimesch et al., 1997). In humans, hippocampal alpha oscillations may be correlated to spatial working memory and likely with long-term memory engrams (Başar et al., 1999). However, in the majority of animal studies, the alpha band has been classified as a part of the theta band (Nerad and Bilkey, 2005).

In previous studies, hippocampal theta oscillation (4–13 Hz) has been shown to possibly modulate memory (Vertes, 2005; Suthana et al., 2012; Luo and Guan, 2018). In addition, hippocampal theta oscillation can be activated by sensory stimuli, which is related to spatial working memory (Givens, 1996). Other animal studies have also reported that DBS of the fornix and medial septal nucleus can improve spatial working memory and enhance hippocampal theta oscillations (Williams and Givens, 2003; Lee et al., 2012; Suthana and Fried, 2014). Thus, improvement in the performance of T-maze behavioral task by CT-DBS was characterized by increased hippocampal theta oscillation, which serve as a potential biomarker for enhancement of spatial working memory.

### CT-DBS Enhanced FC Related to Neurotransmitter Receptors

Increased local theta rhythmic activity synchronized between each two regions of the trisynaptic circuit has indicated that excited projections functionally serve to couple CT and hippocampal connections (Everling et al., 2010; Reinhart and Nguyen, 2019). The FC between the CT and hippocampus consistent with previous studies, including the anatomical mechanisms (Shirvalkar et al., 2006) and neural processing for the modulation of memory (Lee et al., 2017). We examined the synaptic neurotransmitter mechanisms underlying CT-DBS-induced FC changes and performance in the T-maze behavioral task. Our findings revealed increased expression of DRD1 by CT-DBS modulation but decreased expression of DRD2 in the CA1, CA3, and DG.

In rodents, the hippocampus has been extensively studied as one of the brain regions responsible for spatial memory and navigation. The first discovery of synaptic plasticity, known as long-term potentiation (LTP), occurred in the hippocampus, which consolidates the experience to memory (Bliss and Collingridge, 1993; Larkman and Jack, 1995). The trisynaptic circuit has generally been used to study LTP, including the connections from the DG to CA3 and CA3 to CA1 (Madison et al., 1991). In these synapses, dopamine may influence the induction of LTP through specific changes in the levels of cyclic adenosine monophosphate (cAMP), which is a critical regulator of LTP in the hippocampus (Jay, 2003). The activation of DRD2 as a presynaptic receptor serves to inhibit cAMP levels, which in turn inhibits the release of dopamine. DRD1, as a postsynaptic receptor, increases cAMP levels, which subsequently activates the release of dopamine (Nasehi et al., 2018). In addition, the α4-nAChR receptor in the nicotinic cholinergic system plays a role in attention and spatial working memory in the hippocampus, with activation of the α4-nAChR enhancing memory acquisition, consolidation, and information by increasing synaptic modification (Levin et al., 2006).

Increased dopamine and acetylcholine levels enhanced FC in the trisynaptic circuit. DBS may increase DRD1 and decrease DRD2 receptor expressions, contributing to increased activity of the dopamine synthesis enzyme and increasing dopamine neurotransmission in the hippocampus. Moreover, another study confirmed that increased release of acetylcholine enhances spatial working memory (Fadda et al., 1996). The application of CT-DBS contributed to the regulation of DRD1, DRD2 and α4-nAChR receptor expressions in the CA1, CA3 and DG, indicating that enhancement of performance in the T-maze behavioral task may be due to decreasing DRD2 activity and increasing DRD1and α4-nAChR activities. Thus, CT-DBS potentially modulates synaptic plasticity by altering the expression of dopaminergic and cholinergic receptors, resulting in the enhancement of FC in the trisynaptic circuit for spatial working memory.

## Conclusion

In this study, CT-DBS revealed the enhancement of T-maze behavioral task performance, which was related to spatial working memory and elevated hippocampal theta oscillations. Meanwhile, CT-DBS promoted the FC between the CT and subregions (DG, CA3, and CA1) in the hippocampus due to neuroplasticity modulation (possibly due to altered expressions of the DRD1, DRD2, and α4-nAChR receptors). Therefore, our data may provide insights into the importance of CT-DBS in novel therapeutic approaches to improve memory.

## Data Availability Statement

The datasets generated for this study are available on request to the corresponding author.

## Ethics Statement

The animal study was reviewed and approved by the Institutional Animal Care and Use Committee of Tzu Chi General Hospital (IACUC Approval No. 106-35).

## Author Contributions

C-WC, S-HL, S-YC, and Y-YC designed the project and organized the entire research. Y-CL, S-HY, and H-CL conceived the experiments. Y-CL, T-CL, S-JL, CH, VR, and C-HK conducted the experiments. C-WC, S-HY, T-CL, and S-JL analyzed the results. H-CL, Y-JC, Y-CC, and C-WL performed the immunohistochemistry and Western blot studies. C-WC, CH, S-HL, and Y-YC wrote the manuscript. All authors discussed the results and reviewed on the manuscript.

## Conflict of Interest

The authors declare that the research was conducted in the absence of any commercial or financial relationships that could be construed as a potential conflict of interest.

## Footnotes

1 http://imagej.nih.gov/ij/
