# Supplementary material for: Modulation of Theta-Band Local Field Potential Oscillations Across Brain Networks With Central Thalamic Deep Brain Stimulation to Enhance Spatial Working Memory
Source: Front Neurosci. 2019 Nov 26;13:1269. doi: 10.3389/fnins.2019.01269 (PMC6988804; doi:10.3389/fnins.2019.01269)
Supplement: Supplementary file 1 [file Table_1.docx]

Supplementary Material

Modulation of theta-band local field potential oscillations across brain networks with central thalamic deep brain stimulation to enhance spatial working memory

Ching-Wen Chang^1, †^, Yu-Chun Lo^2,^ ^†^, Sheng-Huang Lin^3, 4, *^, Shih-Hung Yang^5^, Hui-Ching Lin^6^, Ting-Chun Lin^1^, Ssu-Ju Li^1^, Christine Chin-Jung Hsieh^1, 7^, Vina Ro^1^, Yueh-Jung Chung^6^, Yun-Chi Chang^6^, Chi-Wei Lee^2,6^, Chao-Hung Kuo^1, 8, 9^, Shin-Yuan Chen^10, 11^, and You-Yin Chen ^1, 2, 7, *^

^1^ Department of Biomedical Engineering, National Yang Ming University, No.155, Sec.2, Linong St., Taipei, Taiwan 11221, R.O.C.

^2^ The Ph.D. Program for Neural Regenerative Medicine, College of Medical Science and Technology, Taipei Medical University, No. 250 Wu-Xing St., Taipei 11031, Taiwan, R.O.C.

^3^ Department of Neurology, Hualien Tzu Chi Hospital, Buddhist Tzu Chi Medical Foundation, No. 707, Sec. 3, Chung Yang Rd., Hualien 97002, Taiwan, R.O.C.

^4^ Department of Neurology, School of Medicine, Tzu Chi University, No. 701, Sec. 3, Zhongyang Rd., Hualien 97004, Taiwan, R.O.C.

5 Department of Mechanical Engineering, National Cheng Kung University, No. 1 University Rd., Tainan, Taiwan 70101, R.O.C.

^6^ Department and Institute of Physiology, National Yang Ming University, No.155, Sec.2, Linong St., Taipei, Taiwan 11221, R.O.C.

^7^ Taiwan International Graduate Program in Interdisciplinary Neuroscience, National Yang Ming University and Academia Sinica, No.128, Sec. 2, Academia Rd., Taipei, Taiwan 11529, R.O.C.

^8^ Department of Neurosurgery, Neurological Institute, Taipei Veterans General Hospital, No.201, Sec. 2, Shipai Rd., Taipei, Taiwan11217, R.O.C.

^9^ Department of Neurological Surgery, University of Washington, No.1959 NE Pacific St., Seattle, WA 98195-6470, U.S.A.

^10^ Department of Neurosurgery, Hualien Tzu Chi Hospital, Buddhist Tzu Chi Medical Foundation, No. 707, Sec. 3, Chung Yang Rd., Hualien 97002, Taiwan, R.O.C.

^11^ Department of Surgery, School of Medicine, Tzu Chi University, No. 701, Sec. 3, Zhongyang Rd., Hualien 97004, Taiwan, R.O.C.

^†^These authors have contributed equally to this work.

^*^Correspondence should be addressed to the following:

Department of Neurology, Hualien Tzu Chi Hospital, Buddhist Tzu Chi Medical Foundation, No. 707, Sec. 3, Chung Yang Rd., Hualien 97002, Taiwan, R.O.C.

Department of Neurology, School of Medicine, Tzu Chi University, No. 701, Sec. 3, Zhongyang Rd., Hualien 97004, Taiwan, R.O.C.

E-mail: shlin355@gmail.com

You-Yin Chen, Department of Biomedical Engineering, National Yang Ming University, No.155, Sec.2, Linong St., Taipei, Taiwan 11221, R.O.C.

E-mail: irradiance@so-net.net.tw

*Note 1. CT-DBS-evoked LFP oscillation changes*

The LFP oscillatory changes before and after CT-DBS in the DBS *wo.* T-maze group showed the significant increase in the LFP theta- and alpha-band oscillations in the CT (theta: 270.27 ± 6.42% [^***^*p* = 0.00046] and alpha: 230.29 ± 5.42% [^***^*p* = 0.00076]), DG (theta: 145.26 ± 6.41% [^*^*p* = 0.03752] and alpha: 140.91 ± 5.42% [^*^*p* = 0.02371]), CA1 (theta: 220.12 ± 6.42% [^**^*p* = 0.00379] and alpha: 210.29 ± 5.44% [^**^*p* = 0.00493]), and CA3 (theta: 230.54 ± 6.41% [^***^*p* = 0.00024] and alpha: 210.41 ± 5.43%, [^***^*p* = 0.00077]) as shown in **Figure S1**. In addition, the measurements of the corresponding effect sizes and the powers of neural oscillation were presented in **Table S1**. With the additional evidence, it was concluded that theta oscillation was increased by CT-DBS.

**Figure S1**. PSD analysis in the DBS *wo.* T-maze group in these brain regions: CT, DG, CA1, and CA3. (A) Following 7-day CT-DBS, theta and alpha-band LFP oscillations also showed significant increases in the CT, DG, CA1, and CA3 compared with those before CT-DBS (on 8^th^ day). (B) The normalized percentage of the LFP PSD was calculated as the ratio of the original PSD before CT-DBS to that after CT-DBS without the T-maze task in the CT, DG, CA1, and CA3. In the DBS *wo.* T-maze group, LFP PSD showed significant enhancements for the theta and alpha bands in the CT, DG, CA1 and CA3 by CT-DBS. ^*^, ^**,^ and ^***^ indicate significantly increased strength of normalized LFP PSD with *P* < 0.05, *P* < 0.01, and *P* < 0.001, respectively, relative to before CT-DBS, analyzed using the Wilcoxon signed-rank test (mean ± SEM). *Abbreviation: LFP: local field potential; PSD: power spectral density; CT: central thalamus; CA1: cornu ammonis region 1; CA3: cornu ammonis region 3; DG: dentate gyrus.*

**Table S1**. The calculations of effect sizes and powers of different frequency bands of neural oscillation in the DBS *wo.* T-maze group.

| *Brain region* | *Frequency band of LFP* | *Effect size* | *Power* |
| --- | --- | --- | --- |
| CT | Delta | 0.285 | 0.358 |
|  | Theta | 2.579 | 0.989 |
|  | Alpha | 1.691 | 0.927 |
|  | Beta | 0.174 | 0.293 |
| DG | Delta | 0.307 | 0.371 |
|  | Theta | 1.359 | 0.861 |
|  | Alpha | 1.287 | 0.841 |
|  | Beta | 0.166 | 0.289 |
| CA1 | Delta | 0.441 | 0.451 |
|  | Theta | 1.317 | 0.849 |
|  | Alpha | 1.562 | 0.905 |
|  | Beta | 0.183 | 0.298 |
| CA3 | Delta | 0.028 | 0.214 |
|  | Theta | 1.359 | 0.861 |
|  | Alpha | 1.663 | 0.923 |
|  | Beta | 0.471 | 0.469 |

*Note 2. Frequency spectrum analysis*

All multichannel local field potentials (LFPs) were first bandpass filtered at a frequency of 0.5–55 Hz with a second-order IIR Butterworth digital filter, downsampled at a frequency of 200 Hz, and then sliced into 10-s windows. The power spectral density (PSD) of LFPs was computed using fast Fourier transform using the Welch spectral estimator (Welch, 1967) and a Hamming window of 2 s with 50% overlap and 1-Hz resolution (Buzsáki, Anastassiou, & Koch, 2012; Jia, Smith, & Kohn, 2011). Each recording session was detrended to remove any slow DC components and zero padded to increase frequency resolution. The PSD of LFP oscillations in each region in the DBS (or sham control) group was calculated compared with that at the baseline before CT-DBS (on the 8^th^ day).

Following CT-DBS, apparent peaks in the theta and alpha bands were found on the PSD analysis of LFPs in the CT, DG, CA1, and CA3 in the DBS group (**Figure S2(A)**). However, no significant changes were found on PSD analysis of LFPs in the CT, DG, CA1, and CA3 between their corresponding baseline and T-maze behavioral task training (**Figure S2(B)**).


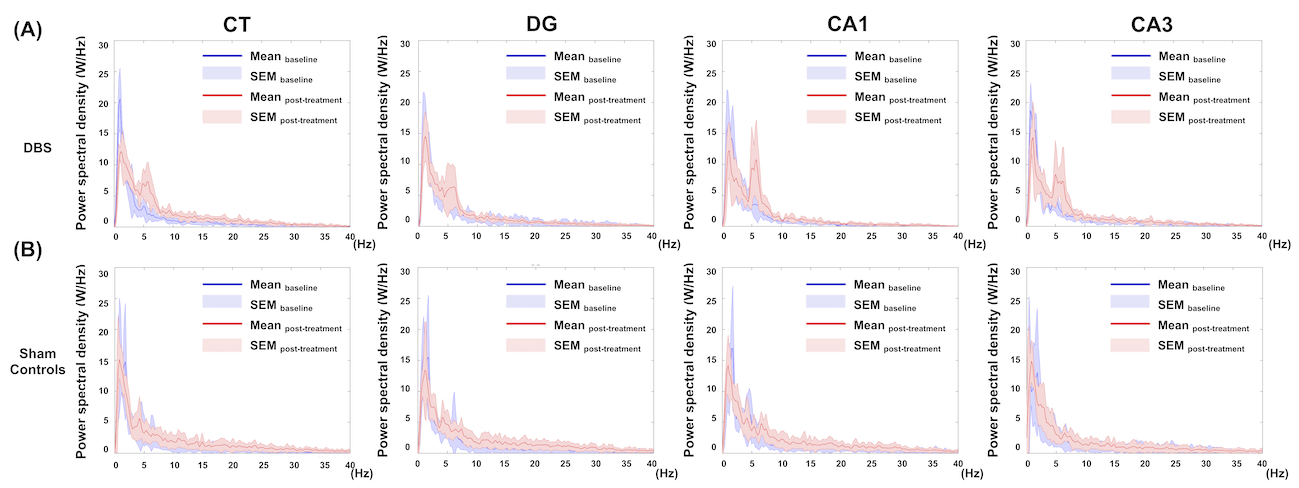


**Figure S2**. PSD analysis in the DBS group and sham controls in these brain regions: CT, DG, CA1, and CA3. (A) Following 7-day CT-DBS, theta and alpha-band LFP oscillations showed significant increases in the CT, DG, CA1, and CA3 compared with those before CT-DBS (on 8^th^ day). (B) There were no significant differences in terms of LFP PSD in the sham controls between their corresponding baseline and T-maze behavioral task training values in four brain regions. The blue line denotes the mean PSD at baseline recorded before CT-DBS (8^th^ day), whereas the red line denotes the mean post-treatment PSD recorded after CT-DBS (or sham controls without CT-DBS) (16^th^ day).

*Note 3. The measurements of effect size and the power for statistical analysis*

All results of behavior performance, neural oscillations, functional brain connectivity, and western blot analysis were calculated the effect size by *Cohen’s d* equation and performed by the power of statistical analysis as shown in **Table S2**, **Table S3**, **Table S4**, and **Table S5**, respectively. *Cohen’s d* was usually used to estimate the sample size for statistical analysis, and the effect size measurements represented the size of the experimental effect (Cohen, 1992; Shavelson, 1988). The *d* measurements from 0.2 to 0.5 mean the small effect size, between 0.5 to 0.8 mean medium effect size, and more than 0.8 means large effect size (Rosnow & Rosenthal, 1996; Rosnow, Rosenthal, & Rubin, 2000). There was decreasing probability of type II error since the power increased (Cacioppo, Tassinary, & Berntson, 2007).

**Table S2**. The effect sizes and powers in latency time and spatial working memory index.

| *Behavioral performance* | *Day* | *Effect size* | *Power* |
| --- | --- | --- | --- |
| Latency time | 09 | 0.289 | 0.267 |
|  | 10 | 0.060 | 0.203 |
|  | 11 | 0.509 | 0.381 |
|  | 12 | 0.559 | 0.411 |
|  | 13 | 2.158 | 0.962 |
|  | 14 | 2.237 | 0.968 |
|  | 15 | 4.338 | 0.999 |
| SWMI | 09 | 0.000 | 0.200 |
|  | 10 | 0.182 | 0.228 |
|  | 11 | 0.425 | 0.334 |
|  | 12 | 1.788 | 0.917 |
|  | 13 | 2.958 | 0.994 |
|  | 14 | 2.731 | 0.989 |
|  | 15 | 7.020 | 0.999 |

**Table S3**. The effect sizes and powers of different frequency bands of neural oscillations in sham controls and the DBS group.

| *Brain region* | *Frequency band of LFP* | *Sham controls* | | *DBS group* | |
| --- | --- | --- | --- | --- | --- |
|  |  | ***Effect size*** | ***Power*** | ***Effect size*** | ***Power*** |
| CT | Delta | 0.317 | 0.279 | 0.437 | 0.340 |
|  | Theta | 1.647 | 0.889 | 3.542 | 0.998 |
|  | Alpha | 1.786 | 0.916 | 3.415 | 0.998 |
|  | Beta | 0.470 | 0.359 | 0.314 | 0.278 |
| DG | Delta | 0.262 | 0.256 | 0.339 | 0.290 |
|  | Theta | 0.546 | 0.403 | 2.167 | 0.962 |
|  | Alpha | 1.047 | 0.685 | 3.629 | 0.998 |
|  | Beta | 0.358 | 0.299 | 0.109 | 0.210 |
| CA1 | Delta | 0.230 | 0.244 | 1.210 | 0.757 |
|  | Theta | 0.076 | 0.205 | 4.914 | 0.999 |
|  | Alpha | 0.611 | 0.442 | 5.055 | 0.999 |
|  | Beta | 0.790 | 0.548 | 0.527 | 0.392 |
| CA3 | Delta | 0.972 | 0.648 | 0.922 | 0.622 |
|  | Theta | 0.309 | 0.276 | 7.262 | 0.999 |
|  | Alpha | 0.694 | 0.492 | 1.719 | 0.904 |
|  | Beta | 0.300 | 0.272 | 0.258 | 0.254 |

**Table S4**. The effect sizes and powers of functional connectivity between spatially distinct brain regions in sham controls and the DBS group.

| *Frequency band of LFP* | *Brain region pairs* | *Sham controls* | | *DBS group* | |
| --- | --- | --- | --- | --- | --- |
|  |  | ***Effect size*** | ***Power*** | ***Effect size*** | ***Power*** |
| Theta band | CT-DG | 1.247 | 0.771 | 2.657 | 0.987 |
|  | CT-CA1 | 0.078 | 0.205 | 2.594 | 0.985 |
|  | CT-CA3 | 0.392 | 0.316 | 0.822 | 0.566 |
|  | DG-CA1 | 0.393 | 0.317 | 0.951 | 0.637 |
|  | DG-CA3 | 0.295 | 0.269 | 2.542 | 0.984 |
|  | CA1-CA3 | 0.134 | 0.215 | 5.119 | 0.999 |
| Alpha band | CT-DG | 0.224 | 0.241 | 3.981 | 0.999 |
|  | CT-CA1 | 0.179 | 0.227 | 1.729 | 0.906 |
|  | CT-CA3 | 0.468 | 0.358 | 0.804 | 0.556 |
|  | DG-CA1 | 0.251 | 0.251 | 0.396 | 0.318 |
|  | DG-CA3 | 0.436 | 0.340 | 1.385 | 0.820 |
|  | CA1-CA3 | 0.058 | 0.202 | 2.259 | 0.969 |
| Delta band | CT-DG | 0.881 | 0.599 | 0.240 | 0.247 |
|  | CT-CA1 | 0.472 | 0.359 | 0.759 | 0.529 |
|  | CT-CA3 | 0.493 | 0.372 | 0.176 | 0.226 |
|  | DG-CA1 | 0.743 | 0.521 | 0.380 | 0.310 |
|  | DG-CA3 | 0.315 | 0.278 | 0.309 | 0.276 |
|  | CA1-CA3 | 0.172 | 0.225 | 0.125 | 0.213 |
| Beta band | CT-DG | 0.725 | 0.510 | 1.340 | 0.805 |
|  | CT-CA1 | 0.459 | 0.353 | 1.392 | 0.822 |
|  | CT-CA3 | 0.466 | 0.357 | 0.344 | 0.292 |
|  | DG-CA1 | 0.074 | 0.205 | 0.456 | 0.351 |
|  | DG-CA3 | 0.597 | 0.433 | 0.142 | 0.217 |
|  | CA1-CA3 | 0.127 | 0.214 | 0.472 | 0.360 |

**Table S5**. The effect sizes and powers for the normalized protein expression levels of DRD1, DRD2 and α4-nAChR in different brain regions. Each sample was presented as a ratio relative to the sham controls.

| *Brain region* | *Receptor protein* | | *Effect size* | *Power* |
| --- | --- | --- | --- | --- |
| CA1 | | DRD1 | 2.500 | 0.982 |
|  |  | DRD2 | 2.728 | 0.989 |
|  |  | α4-nAChR | 2.116 | 0.958 |
| CA3 | | DRD1 | 2.343 | 0.974 |
|  |  | DRD2 | 5.567 | 0.999 |
|  |  | α4-nAChR | 1.887 | 0.951 |
| DG | | DRD1 | 2.978 | 0.994 |
|  |  | DRD2 | 3.707 | 0.998 |
|  |  | α4-nAChR | 3.176 | 0.996 |

*Note 4. Comparison of brain connectivity in beta and delta bands: before (baseline) vs. after CT-DBS*

**Figure S3**. Statistical FC changes before (baseline) and after CT-DBS (or sham stimulation) in each group. FC between pairs of brain regions (CT–DG, CT–CA1, CT–CA3, DG–CA1, DG–CA3, and CA1–CA3) in the (A) sham controls and (B) DBS group. There were no significant differences in terms of FC changes with delta- and beta-band LFP coherences before (baseline) and after CT-DBS (or sham stimulation) for each group (Wilcoxon signed-rank test). All data are presented as the mean ± SEM.

**Reference**

Buzsáki, G., Anastassiou, C. A., & Koch, C. (2012). The origin of extracellular fields and currents — EEG, ECoG, LFP and spikes. *Nature Reviews Neuroscience, 13*(6), 407-420.

Cacioppo, J. T., Tassinary, L. G., & Berntson, G. (2007). *Handbook of psychophysiology*: Cambridge University Press.

Cohen, J. (1992). A power primer. *Psychological Bulletin, 112*(1), 155-159. doi:10.1037/0033-2909.112.1.155

Jia, X., Smith, M. A., & Kohn, A. (2011). Stimulus selectivity and spatial coherence of gamma components of the local field potential. *The Journal of Neuroscience, 31*(25), 9390-9403. doi:10.1523/jneurosci.0645-11.2011

Rosnow, R. L., & Rosenthal, R. (1996). Computing contrasts, effect sizes, and counternulls on other people's published data: General procedures for research consumers. *Psychological methods, 1*(4), 331.

Rosnow, R. L., Rosenthal, R., & Rubin, D. B. (2000). Contrasts and correlations in effect-size estimation. *Psychological science, 11*(6), 446-453.

Shavelson, R. J. (1988). Statistical reasoning for the behavioral sciences.

Welch, P. (1967). The use of fast Fourier transform for the estimation of power spectra: A method based on time averaging over short, modified periodograms. *IEEE Transactions on Audio and Electroacoustics, 15*(2), 70-73. doi:10.1109/TAU.1967.1161901
